# Pulmonary Follow-Up Imaging in Cartilage-Hair Hypoplasia: a Prospective Cohort Study

**DOI:** 10.1007/s10875-021-01007-5

**Published:** 2021-03-05

**Authors:** Svetlana Vakkilainen, Paula Klemetti, Timi Martelius, Mikko JR Seppänen, Outi Mäkitie, Sanna Toiviainen-Salo

**Affiliations:** 1 Children’s Hospital, Pediatric Research Center, University of Helsinki and Helsinki University Hospital, Stenbäckinkatu 9, P.O. Box 347, 00029 HUS Helsinki, Finland; 2 Institute of Genetics, Folkhälsan Research Center, Haartmaninkatu 8, 00290 Helsinki, Finland; 3 Research Program for Clinical and Molecular Metabolism, Faculty of Medicine, University of Helsinki, Haartmaninkatu 8, 00014 Helsinki, Finland; 4 Adult Immunodeficiency Unit, Inflammation Center/Infectious Diseases, University of Helsinki and HUS Helsinki University Hospital, Helsinki, Finland; 5 Rare Disease and Pediatric Research Centers, Hospital for Children and Adolescents, Helsinki, Finland; 6 Department of Molecular Medicine and Surgery and Center for Molecular Medicine, Karolinska Institutet, Visionsgatan 18, 171 76 Stockholm, Sweden; 7 Department of Clinical Genetics, Karolinska University Hospital, Visionsgatan 18, 171 76 Stockholm, Sweden; 8 Medical Imaging Center, Pediatric Radiology, University of Helsinki and HUS Helsinki University Hospital, Helsinki, Finland

**Keywords:** CHH, combined immunodeficiency, lung imaging, MRI, pulmonary complications, RMRP

## Abstract

Cartilage-hair hypoplasia is a syndromic immunodeficiency with short stature, chondrodysplasia, and variable degree of immune dysfunction. Patients with cartilage-hair hypoplasia are prone to recurrent respiratory tract infections, and the prevalence of bronchiectasis ranges from 29 to 52%. Pulmonary complications contribute significantly to the mortality; therefore, regular lung imaging is essential. However, the optimal schedule for repeated lung imaging remains unestablished. We determined the rate and correlates of progression of structural lung changes in a prospectively followed cohort of 16 patients with cartilage-hair hypoplasia. We analyzed clinical, laboratory, and pulmonary functional testing data and performed lung magnetic resonance imaging at a median interval of 6.8 years since previous imaging. Imaging findings remained identical or improved due to disappearance of inflammatory changes in all evaluated patients. Patients with subtle signs of bronchiectasis on imaging tended to have low immunoglobulin M levels, as well as suffered from pneumonia during the follow-up. In conclusion, our results suggest slow if any development of bronchiectasis in selected subjects with cartilage-hair hypoplasia.

**Supplementary Information:**

The online version contains supplementary material available at 10.1007/s10875-021-01007-5.

## Introduction

Cartilage-hair hypoplasia (CHH, MIM # 250250) is a syndromic immunodeficiency characterized by short stature, chondrodysplasia, and variable degree of immune dysfunction [1, 2]. CHH is caused by pathogenic variants in the *RMRP* gene, encoding the untranslated RNA component of the mitochondrial endoribonuclease RNase MRP [3]. The pathogenesis of immunodeficiency in CHH is complex and includes defective cell cycle, impaired telomere maintenance, and altered gene regulation [4–6]. Clinically, immune dysfunction in CHH leads to increased susceptibility to infections, mostly recurrent upper and lower respiratory tract infections, autoimmunity, and increased incidence of malignancy [7–9]. Bronchiectasis is common, with prevalence ranging from 29% in unselected patients and up to 52% in those with chronic respiratory symptoms [10, 11]. Pulmonary infections and bronchiectasis contribute significantly to the increased mortality [12]. Therefore, regular pulmonary imaging is essential, and bronchiectasis can reliably be followed up with lung magnetic resonance imaging (MRI), thus sparing patients from ionizing radiation [10]. However, the rate and determinants of progression of pulmonary changes and the optimal schedule for follow-up imaging in CHH remain unestablished.

## Patients and Methods

We have followed a cohort of 16 patients with CHH, including all those patients for whom lung MRI had been performed in our previous study [10]. We aimed to (1) evaluate the progression of bronchiectasis at MRI, (2) analyze the pattern of respiratory infections, and (3) correlate clinical, laboratory, and pulmonary functional testing data with imaging findings. Informed consent was obtained from all patients and the study was approved by the Institutional Ethics Committee at the Helsinki University Hospital. The imaging equipment, protocol, and bronchiectasis scoring were identical to previously published; however, we used no contrast media [10].

Lung MRI was performed with a 1.5 T scanner (Achieva, Philips Medical System, Best, The Netherlands) with a same scanner and identical imaging protocol used in the previously published study, except for the contrast agent administration and the contrast-enhanced sequences. Approximate imaging time for MRI was 20 min. The protocol included the following sequences: (i) coronal breath-hold single-shot turbo spin echo (field of view (FOV) 315, time of repetition (TR) 786, time to echo (TE) 73.2, slice thickness 6 mm, spacing 4 mm), (ii) coronal and (iii) axial breath-hold 3-dimensional fast field echo (FOV 290, TR 3, TE 0.9, slice thickness 8 mm, spacing 4 mm), (iv) coronal balanced fast field echo (FOV 265, TR 3.4, TE 1.7, slice thickness 4 mm, spacing 2 mm), (v) axial fat-saturated T2-weighted (FOV 260, TR 4305, TE 60, slice thickness 6 mm, spacing 6.5), and (vi) axial and (vii) coronal respiratory- and cardiac-triggered T2-weighted turbo spin echo (FOV 280–327, TR 1500–1798, TE 90–100, slice thickness 5 and 8 mm), in (viii) inspirium and in (ix) expirium coronal balanced fast field echo (FOV 265, TR 3.4, TE 1.7, slice thickness 4 mm, spacing 2 mm).

All MRI studies were analyzed and scored by an experienced radiologist in random order in PACS workstation (Agfa Impax 6.5.2.2101). Scoring was performed using the modified Helbich (Bhalla) system [13]. Nine parameters were taken into account when evaluating high-resolution computed tomography (HRCT) and MRI images and a maximum possible score was 27 points. The score covered nine categories of changes, each scored from zero to three: (1) severity of bronchiectasis, (2) severity of peribronchial wall thickening, (3) extent of bronchiectasis, (4) extent of mucus plugging, (5) extent of sacculation or abscesses, (6) generation of bronchial division involved, (7) severity of bullae, (8) severity of emphysema, and (9) severity of collapse or consolidation. Score ≥ 7 was chosen as cutoff value for bronchiectasis.

We used Chi-square test to search for correlates between MRI score and clinical and laboratory variables. Statistical analyses were performed with IBM SPSS version 25 software.

## Results

Table 1 describes clinical characteristics of the study patients. All patients were homozygous (*n* = 15) or compound heterozygous (*n* = 1, n.263G > T) for the n.71A > G *RMRP* mutation. Adult height in the majority of patients (15/16) represented growth between the 10 h and the 90th percentile on CHH-specific growth curves, while patient 10 demonstrated severe growth failure below the 10th percentile [14]. The cohort was diverse in their age (median 41 years, range 20–68 years, at the time of latest MRI) and in their clinical immunodeficiency phenotype (from asymptomatic to combined). The most common clinical symptoms of immunodeficiency in this cohort were recurrent rhinosinusitis, refractory mucocutaneous warts, as well as recurrent acute otitis media, which were reported in 11/16 (69%), 8/16 (50%), and 7/16 (44%) patients during lifetime, respectively. However, only two patients had ever received prophylactic antibiotics for recurrent respiratory tract infections, both in adulthood.

**Table 1:** Clinical characteristics of the study patients

Patient	Age group at initial lung imaging, years	During lifetime				Period between repeated MRI		
		Physician-diagnosed asthma	Regular ICS	Prophylactic antibiotics for Rec infections	Clinical symptoms of immunodeficiency	Pneumonia	Other infections	IGRT
1	13–18	No	No	In adulthood	Rec OM, refractory warts, autoimmunity	No	Sin once	Yes
2	13–18	Yes, in childhood	No	No	None	No	No	No
3	13–18	No	No	No	Rec OM and Sin, refractory warts	No	No	No
4	19–24	No	No	No	Refractory warts	No	No	No
5	19–24	Yes, in adolescence	Yes	No	Rec OM and Sin, refractory warts	No	OM and Sin twice	No
6	24–29	Yes, in adulthood	Yes	No	Rec OM and Sin, refractory warts	Yes, twice	No	Yes, for 1 year
7	30–35	No	No	No	Rec Sin	No	Sin once	No
8	30–35	No	No	No	Rec Sin	No	No	No
9	36–41	No	No	No	Rec Sin, refractory warts	Nos	Rec Sin	No
10	36–41	No	No	No	Rec OM and Sin, boils, refractory warts and molluscum	No	Rec OM and Sin	Yes
11	36–41	No	No	No	Rec OM and Sin	No	Rec Sin	No
12	36–41	Yes, in adulthood	Yes	No	Rec pneumonia, OM and Sin, severe varicella	No	Sin thrice	No
13	54–59	No	No	No	None	No	Sin thrice	No
14	60–65	No	No	In late adulthood	Rec pneumonia and Sin	Yes, once	Sin thrice	No
15	60–65	No	No	No	Rec Sin, refractory warts	No	Rec Sin	No
16	66–71	Yes, in late adulthood	Yes	No	None	No	No	No

*ICS* inhaled corticosteroids, *IGRT* immunoglobulin replacement therapy, *n/a* not available, *OM* acute otitis media, *Rec* recurrent, *Sin* rhinosinusitis

All patients were homozygous for *RMRP* variant n.71A > G, except patient 7 who was compound heterozygous (n.71A > G/n.263G > T)

**Fig. 1:**
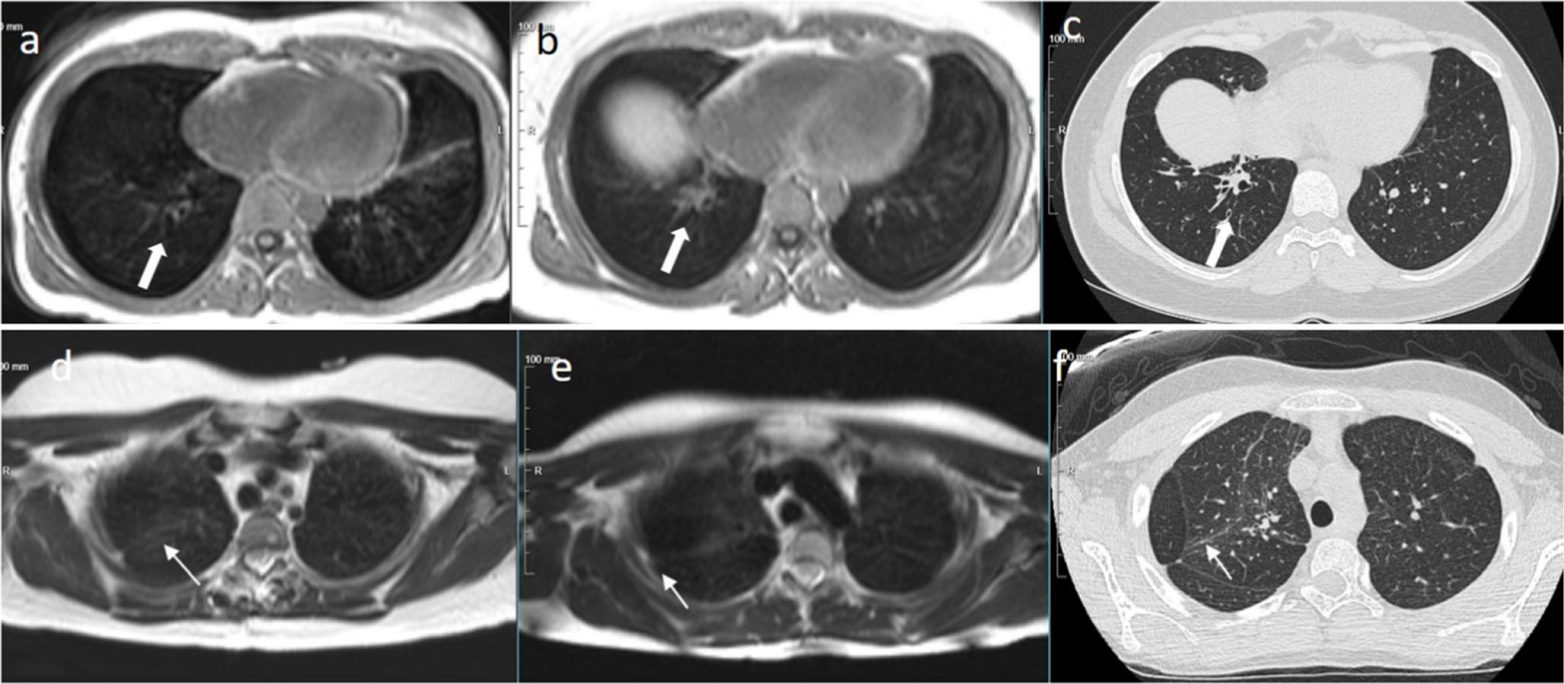
Pulmonary imaging of two study participants. Upper row: patient 6 with stable bronchiectasis (thick arrows) of the lower lobes; **a** the recent MRI (axial 3DFFE sequence) and 6 years earlier obtained, **b** baseline MRI (axial 3DFFE sequence), and **c** HRCT. Lower row: patient 2 with apical pleural thickening, parenchymal stranding and scarring (thin arrows), but no bronchiectasis. No progression of findings from baseline in HRCT or MRI imaging; **d** the recent MRI (axial T2 TSE sequence) and 6 years earlier obtained, **e** baseline MRI, and **f** HRCT

Table 2 demonstrates the results of pulmonary imaging and lung functional testing in the study patients. Repeated MRI was performed in 14/16 patients: at a median interval of 6.8 years (range 5.9–8.3 years). Patient 16 had deceased due to an unknown cause and for patient 13 logistic issues prevented imaging. MRI bronchiectasis scores remained identical to previous assessments in 11 patients and improved in three patients due to the disappearance of acute inflammatory changes. Figure 1 demonstrates the nonprogression of the structural changes in two patients. Of three patients with bronchiectasis (bronchiectasis score ≥ 7 points) detected already in their initial MRI, two did not undergo repeated imaging while the third cleared inflammatory changes (score dropped to <7). Three patients had also undergone repeated lung HRCT at the discretion of the treating physicians, and the results were compatible with repeated MRI, showing no progression of bronchiectasis. Lung diffusion capacity testing in three patients demonstrated normal results.

**Table 2:** Comparison of the lung magnetic resonance imaging (MRI) bronchiectasis (BE) scores of the study patients, as well as the results of lung diffusion capacity testing and high-resolution computed tomography (HRCT) lung imaging performed in-between repeated MRI

Patient	Age group at initial MRI, years	Previous MRI BE score	Interval between repeated MRI, years	Repeated MRI BE score	Lung diffusion capacity	Lung HRCT after initial MRI
1	13–18	0	8.3	0	n/a	5 years later, no BE
2	13–18	3	6.8	1	n/a	3 years later, no progression
3	13–18	0	6.6	0	Normal	5 years later, no BE
4	19–24	0	7.4	0	n/a	Not performed
5	19–24	0	7.0	0	Normal	Not performed
6	24–29	6	6.9	6	n/a	Not performed
7	30–35	3	6.7	0	n/a	Not performed
8	30–35	8	7.0	5	n/a	Not performed
9	36–41	0	7.0	0	n/a	Not performed
10	36–41	0	6.7	0	n/a	Not performed
11	36–41	0	5.9	0	Normal	Not performed
12	36–41	0	6.8	0	n/a	Not performed
13^a^	54–59	7	NA	NA	n/a	Not performed
14	60–65	4	6.7	4	n/a	Not performed
15	60–65	4	6.6	4	n/a	Not performed
16^a^	66–71	9	NA	NA	n/a	Not performed

n/a not available, NA not applicable

^a^Repeated lung imaging could not be arranged

A cutoff value for bronchiectasis was ≥7 points

Two patients had acquired pneumonia during follow-up: patient 6 was treated at home, while patient 14 required hospitalization (Table 1). Both had some structural lung changes on initial imaging (6 and 4 points, respectively), which did not progress during follow-up. Our cohort was diverse in terms of other respiratory tract infections: while some patients remained infection-free during follow-up, others suffered from recurrent rhinosinusitis and otitis media. Five out of 16 participants (31%) had been previously diagnosed with asthma and four of them had been receiving regular inhaled corticosteroids starting from adolescence (*n* = 1) or adulthood (*n* = 3). The prevalence of asthma in our cohort was similar to the prevalence of 23% within a larger (*n* = 104) cohort of Finnish patients with CHH (*p* value in the Fisher exact test 0.53) [8]. None of the patients diagnosed with asthma developed novel or progressive previous lung changes on follow-up imaging. None of the patients received antibiotic prophylaxis during follow-up. Two patients received immunoglobulin (Ig) replacement therapy for the duration of follow-up. The indications for therapy had been recurrent infections, deficiency of one or more IgG subclasses, and specific antipolysaccharide antibody deficiency; none of the study participants had decreased total IgG. These two patients did not experience pneumonia or develop structural lung changes during follow-up. Neither did six other patients with zero bronchiectasis scores, who had not received Ig replacement.

The lymphocyte profile of study participants was variable, showing normal or low counts of measured lymphocyte subpopulations, but no correlation with lung changes (Table 3, *Online Resource 1*). The most consistent laboratory finding was the decreased number of recent thymic emigrants, B cells, and CD4+ T cells, detected in 9/16 (56%), 8/16 (50%), and 7/16 (44%) of patients, respectively. The unexpectedly high proportion of patients with normal total lymphocyte counts (14/16, 88%) is in contrast to the previously reported high prevalence of lymphopenia in Finnish CHH cohorts [1, 7, 15]. We could not perform the comparison of laboratory parameters between patients with and without bronchiectasis due to the absence of bronchiectasis on the repeated imaging.

**Table 3:** Laboratory characteristics of the study patients

	Total lymphocytes	CD3+	CD4+	CD8+	RTE	CD19+	IgG, g/l	IgA, g/l	IgM, g/l
Reference values	1.5–6.7	0.75–2.76	0.404–1.612	0.22–1.13	0.024–0.824	0.08–0.62	6.8–15	M 0.88–4.84 F 0.52–4.02	M 0.36–2.59 F 0.47–2.84
P 1	Low	Low	Low	Low	Low	Low	Normal	Normal	Normal
P 2	Low	Normal	Normal	Low	Normal	Normal	Normal	Normal	Normal
P 3	Normal	Low	Low	Low	Low	Low	High	Absent	Low
P 4	Normal	Low	Low	Low	Low	Low	Normal	Normal	Normal
P 5	Normal	Low	Low	Low	Low	Normal	Normal	Normal	Normal
P 6	Normal	Normal	Normal	Normal	Low	Low	Normal	Normal	Normal
P 7	Normal	Normal	Normal	Normal	Normal	Low	Normal	Normal	Normal
P 8	Normal	Normal	Low	Normal	Low	Low	Normal	Normal	Low
P 9	Normal	Normal	Normal	Normal	Normal	Low	Normal	Normal	Normal
P 10	Normal	Low	Low	Low	Low	Low	Normal	Normal	Normal
P 11	Normal	Normal	Normal	Normal	Normal	Normal	Normal	Normal	Normal
P 12	Normal	Normal	Normal	Normal	Normal	Normal	Normal	Normal	Normal
P 13	Normal	Low	Low	Normal	Low	Normal	Normal	n/a	n/a
P 14	Normal	Normal	Normal	Normal	Low	Normal	Normal	High	Low
P 15	Normal	Normal	Normal	Normal	Normal	Normal	Normal	High	Low
P 16	Normal	Normal	Normal	Normal	Normal	Normal	Normal	Normal	Normal

F females, M males, n/a not available, P patient, RTE recent thymic emigrants defined as CD3 + CD4 + CD45RA + CD31+ cells

Local laboratory normal reference values were used, in ×10^9^/l for cell counts and in g/l for immunoglobulins (Ig)

There were no correlations between MRI bronchiectasis score and a range of clinical and laboratory parameters, including recurrent rhinosinusitis or otitis media, asthma, low counts of total lymphocytes, CD3+, CD4+, CD8+ T cells, CD19+ B cells, or low IgA levels (*Online Resource 1*). However, out of five patients with bronchiectasis MRI score higher than zero, three had low IgM levels and two had pneumonia during follow-up, compared to single patient with low IgM and no cases of pneumonia in patients with bronchiectasis score of zero (*Χ*^2^(4) = 9.9, *p* 0.042, and *Χ*^2^(4) = 9.6, *p* 0.047, for pneumonia and low IgM, respectively; *Online Resource 1*). These results should be interpreted with caution due to the limited sample size.

In our previous lung imaging study, additional 18 patients underwent HRCT pulmonary imaging, but not lung MRI. All 18 patients are alive at the time of writing. Among these patients, five were diagnosed with bronchiectasis: four with scores of 7 and one with score of 13 (Table 4). Follow-up lung imaging has been performed for four out of eleven patients with bronchiectasis score over zero (Table 4), while for others, follow-up imaging has been deemed clinically unnecessary. The follow-up imaging for these patients was performed outside the study protocol, which prevented the direct comparison of bronchiectasis scores. However, grossly evaluated, no (*n* = 3) or very subtle (*n* = 1) progression of bronchiectasis has been noticed on the follow-up imaging.

**Table 4:** Characteristics and follow-up data of additional 11 CHH patients with bronchiectasis (BE) score > 0 at the initial imaging

Patient	Age group at initial lung imaging, y	Initial lung HRCT BE score	Follow-up lung imaging, progression of BE	Clinical symptoms of immunodeficiency	Physician-diagnosed asthma	Lymphocyte profile	IgA, IgM and IgG levels
17	36–41	7	Not performed	Rec Sin	No	All normal	All normal
18	42–47	5	Not performed	Rec OM and Sin	No	All normal	All normal
19	42–47	7	HRCT 5 y later, no	Rec OM, refractory warts	No	Low RTE	High IgG
20	48–53	2	MRI 3 y later, no	None	No	All normal	All normal
21	54–59	4	HRCT 5 y later, subtle	Rec Sin, severe varicella	In adulthood	Low CD3+, CD4+, CD19+, RTE	Low IgA, IgG
22	60–65	7	Not performed	None	In late adulthood	Low CD3+, CD4+, CD8+, RTE	High IgG
23	60–65	13	HRCT 1.5 y later, no*	None	No	Low RTE	All normal
24	60–65	3	Not performed	Refractory mucocutaneous *Candida* spp. and herpes simplex virus infections	No	All normal	All normal
25	60–65	5	Not performed	None	No	All normal	All normal
26	66–71	7	Not performed	Rec OM and Sin, refractory warts	In late adulthood	Low CD19+, RTE	Low IgM
27	66–71	4	Not performed	None	No	All normal	All normal

*Follow-up HRCT did not detect progression of BE 1.5 years after the initial imaging. Lung biopsy demonstrated chronic bronchiolitis, granulomatous inflammatory changes and organizing pneumonia

HRCT high-resolution computed tomography, Ig immunoglobulin, MRI magnetic resonance imaging, OM acute otitis media, Sin rhinosinusitis, y years

All patients were homozygous for *RMRP* variant n.71A > G, except patients 25 and 27 who were compound heterozygous (n.71A > G/n.263G > T). Local laboratory lowest normal reference values for the peripheral blood lymphocyte subsets were as follows (cells ×10^9^/l): CD3+ 0.75, CD4+ 0.458, CD8+ 0.22, CD19+ 0.08, CD16/56+ 0.08, CD3 + CD4 + CD45RA + CD31+ recent thymic emigrants (RTE) 0.024. None of the patients had ever received immunoglobulin replacement therapy of antibiotic prophylaxis

## Discussion

We have previously reported the high prevalence of bronchiectasis in patients with CHH [10]. Since then, we have followed this reported cohort of patients to determine the rate and correlates of progression of pulmonary changes and the optimal schedule for follow-up imaging. We now describe the clinical and radiological outcomes with a median of 6.8 years of follow-up. The results suggest slow if any development of bronchiectasis in subjects with CHH. We provide evidence for the optimal schedule of follow-up lung imaging to be used by clinicians caring for similar patients.

The factors contributing to the development of bronchiectasis in some, but not all, patients with CHH remain unclear. Despite recurrent respiratory infections and the absence of antimicrobial prophylaxis, bronchiectasis did not develop in our study patients. Both patients who developed pneumonia during the follow-up had subtle structural lung changes that have not, however, progressed. Whether the preexisting pulmonary abnormalities predispose patients to lung infections, or whether the imaging findings and the infections independently reflect the more severe underlying immunodeficiency, remains to be confirmed in further studies.

Noteworthily, only one of our patients had been diagnosed with asthma in childhood, but had not received regular inhaled corticosteroids. Another four patients had been diagnosed with asthma in adolescence or adulthood and had all been treated with regular inhaled corticosteroids. It remains to be explored, whether appropriate management of asthma or asthma-like symptoms in childhood and/or adulthood may prevent the development of bronchiectasis.

We have previously reported higher T cell counts and higher IgG levels in CHH patients with bronchiectasis [10]. Patients in our follow-up cohort had a strikingly low prevalence of lymphopenia, which may underlie the milder course of immunodeficiency and possibly explain the absence of bronchiectasis. The finding of higher prevalence of low IgM levels in patients with subtle bronchiectasis changes is in concordance with reported correlations in patients with common variable immunodeficiency and bronchiectasis [16].

The rate of bronchiectasis development may be variable and influenced by various individual factors. The duration of our study (median 6.8 years of follow-up) may be insufficient to detect pulmonary structural changes. However, our results provide an approximate estimate of imaging schedule for mildly symptomatic patients. Based on our findings, routine pulmonary imaging can be scheduled infrequently, even at longer interval than the 6–8 years used in this study, in patients without bronchiectasis on initial imaging. However, the majority of subjects in our cohort with normal or nonprogressive findings on MRI did not experience pneumonia during follow-up. Therefore, patients with recurrent pneumonias probably warrant more frequent lung imaging. Also, data on patients with established bronchiectasis were limited in our study and no firm recommendations can be derived for the optimal time intervals between follow-up imaging studies.

One important limitation of our study is the genetic homogeneity of the Finnish CHH cohort and the associated restricted phenotype [17]. *RMRP* variants other than the Finnish founder variant n.71A > G can be associated with a more severe phenotype and therefore a different rate of development and/or progression of lung disease [18]. Further collaborative international effort is needed to expand our findings in a larger and genetically more heterogeneous CHH cohort.

The assessment of pulmonary function has been difficult in CHH, due to the absence of specific height-adjusted reference values in adults with short stature. In CHH, the height-related values may be normal even when lung function is diminished, whereas age-related values may be falsely low. For patients with common variable immunodeficiency, diffusion capacity testing does not discriminate patients with bronchiectasis from those without [19], and this may also be true for patients with CHH. In addition, some patients declined or were unable to perform functional testing. Therefore, the use of pulmonary functional testing in our cohort has been limited to three patients precluding interpretation.

HRCT follow-up imaging was not included in our study protocol due to concern about radiation burden; however, HRCT has been performed at the discretion of treating physicians in six patients, all showing none or very subtle progression of bronchiectasis. Although the direct comparison of bronchiectasis scores was impossible due to variability of imaging protocols, for the three patients with available MRI and HRCT follow-up imaging, the results were similar. Coupled with our previous findings [10], this suggests that while lung HRCT remains the gold standard for the initial evaluation of lung changes in patients with immunodeficiency, MRI can be implemented in the follow-up. In conclusion, we provide data on the lung imaging follow-up in patients with CHH. Several limitations should be applied when extrapolating these data to patients with more severe clinical course or established bronchiectasis. However, our results add to the limited knowledge on disease progression and proper management of patients with CHH.

## Supplementary Information

ESM 1 (PDF 91 kb)

## Data Availability

All data generated or analyzed during this study are included in this published article [and its supplementary information files].
