# Supplementary material for: Pulmonary Follow-Up Imaging in Cartilage-Hair Hypoplasia: a Prospective Cohort Study
Source: J Clin Immunol. 2021 Mar 5;41(5):1064–71. doi: 10.1007/s10875-021-01007-5 (PMC8249260; doi:10.1007/s10875-021-01007-5)
Supplement: Supplementary file 1 — (PDF 91 kb) [file 10875_2021_1007_MOESM1_ESM.pdf]

Article title: Pulmonary follow-up imaging in cartilage-hair hypoplasia: a prospective cohort study

Journal name: Journal of Clinical Immunology

Author names: Svetlana Vakkilainen, Paula Klemetti, Timi Martelius, Mikko Seppänen, Outi Mäkitie, Sanna Toiviainen-Salo

Affiliation and e-mail address of the corresponding author:

1 Children's Hospital, Pediatric Research Center, University of Helsinki and Helsinki University Hospital, Stenbäckinkatu 9, Helsinki PO Box 347, 00029, Helsinki, Finland

2 Folkhälsan Research Center, Institute of Genetics, Haartmaninkatu 8, 00290, Helsinki, Finland

3 Research Program for Clinical and Molecular Metabolism, Faculty of Medicine, University of Helsinki, Haartmaninkatu 8, 00014, Helsinki, Finland

* Correspondence: Svetlana Vakkilainen, svetlana.vakkilainen@helsinki.fi, tel +358 9 4711, fax +358 9 471 75 315, Children’s Hospital, Pediatric Research Center, University of Helsinki and Helsinki University Hospital, Stenbäckinkatu 9, PL 347, 00029 HUS, Helsinki, Finland

Online Resourse 1. Correlation of magnetic resonance imaging bronchiectasis scores and various clinical and laboratory features in 14 patients with cartilage-hair hypoplasia, by Chi-square test.

**Feature**	**$X^2(4)$**	**p value**
Physician-diagnosed asthma	6.4	0.173
Pneumonia	9.9	0.042
Recurrent rhinosinusitis	4.8	0.313
Recurrent otitis media	6.0	0.199
Low total lymphocyte count	6.7	0.150
Low CD3+ cell count	4.3	0.364
Low CD4+ cell count	4.9	0.295
Low CD8+ cell count	4.9	0.295
Low count of recent thymic emigrants	2.9	0.577
Low CD19+ cell count	5.8	0.212
Low immunoglobulin A levels	0.6	0.963
Low immunoglobulin M levels	9.6	0.047
